# Attitudes of Spanish Nurses towards Pressure Injury Prevention and Psychometric Characteristics of the Spanish Version of the APuP Instrument

**DOI:** 10.3390/ijerph17228543

**Published:** 2020-11-18

**Authors:** María Dolores López-Franco, Laura Parra-Anguita, Inés María Comino-Sanz, Pedro L. Pancorbo-Hidalgo

**Affiliations:** Department of Nursing, Faculty of Health Sciences, University of Jaén, 23071 Jaén, Spain; lparra@ujaen.es (L.P.-A.); icomino@ujaen.es (I.M.C.-S.); pancorbo@ujaen.es (P.L.P.-H.)

**Keywords:** pressure ulcers, prevention, attitudes, nursing staff, validation studies

## Abstract

The prevention of pressure injuries in hospitalised patients is a critical point of care related to patient safety. Nurses play a key role in pressure injury (PI) prevention, making it important to assess not only their knowledge but also their attitude towards prevention. The main purpose of this study was to translate into Spanish and evaluate the psychometric properties of the Attitude towards Pressure ulcer Prevention instrument (APuP); a secondary aim was to explore the associations of attitude with other factors. A Spanish version was developed through a translation and back-translation procedure. The validation study was conducted on a sample of 438 nursing professionals from four public hospitals in Spain. The analysis includes internal consistency, confirmatory factorial analysis, and construct validity in known groups. The 12-item Spanish version of the APuP fit well in the 5-factor model, with a Cronbach’s alpha of 0.7. The mean APuP score was 39.98, which means a positive attitude. Registered nurses have a slightly better attitude than Assistant nurses. A moderate correlation (R = 0.32) between knowledge and attitude for the prevention of PI was found. As concluded, the Spanish version of the APuP questionnaire is a valid, reliable and useful tool to measure the attitude toward PI prevention in Spanish-speaking contexts. This version has 12 items grouped into 5 factors, and its psychometric properties are similar to those of the original instrument.

## 1. Introduction

In recent years, there has been a debate about the definition, classification and even naming of pressure ulcers (currently, the name “Pressure Injury” (PI) is used). The current definition of PI states that these are areas of localised damage to the skin and the underlying soft tissue, especially in bony prominences or areas in contact with clinical devices. The forces involved in producing the damage are intense pressure or pressure combined with shear [1]. Currently, it is recommended to systematically use one of the classification systems proposed for staging the PI, from the Stage 1 (non-blanchable erythema) to Stage 4 (full thickness of the skin ulcer) [2].

The occurrence of PI is a frequent adverse event in patients admitted to hospitals worldwide. The figures for incidence and prevalence of PI in hospitalised patients largely vary among different countries. A recent systematic review and meta-analysis over 39 published articles on PI prevalence (with a total sample of 2,579,049 patients) has estimated a global PI prevalence of 12.8% (95% CI = 11.8–13.9%) and an incidence of 5.4% per 10,000 patient-days (95% CI = 3.4–7.8) [3]. This systematic review concluded that the burden of PI is still important, with over one in ten adult patients admitted to hospitals affected.

The prevention of PI should be a key objective in healthcare systems. The Rio de Janeiro Declaration on the Prevention of Pressure Ulcers noted the importance of PI prevention as a universal right, the seriousness of the problem for public health, the ethical implications for health professionals, the costs involved for health systems, and the recognition of PI as an adverse event [4]. Since 1988, when Hibbs [5] speculated that as far as 95% of PI could be preventable, this value has been considered as a target for prevention programmes. However, more recent studies have indicated that only 43% of stage 3 and 4 PIs produced were classified as preventable [6].

In any case, prevention is the most cost-effective approach for PI [7]. It is important to consider the concept of the avoidable or unavoidable PI because the scientific discussion is open and the factors involved are complex (ischaemia and reperfusion, pressure and shear over tissues) [8]. It is well known that when preventive interventions are not properly documented and implemented, avoidable PIs occur [9].

### 1.1. Attitudes on PI Prevention

Training healthcare providers in PI prevention is paramount, including assessment, identification, classification, use of equipment, skin care and preventive interventions. The use of an evidence-based risk assessment tool has led to a decrease in the prevalence of hospital-acquired PIs from 7.5% to less than 4% in Saudi Arabia [10]. A specific training programme developed over 4 years in Australia has resulted in a decrease in the prevalence of PI from 6.7 to 1.9% [11].

Among health care providers, nurses play a key role in PI prevention. Therefore, to successfully implement a preventive programme in health care facilities, it is necessary to consider the knowledge they have, using properly validated questionnaires [12,13], but also to determine attitudes [14]. Azjen defined an attitude as “*a disposition to respond favourably or unfavourably to an object, person, institution, or event*”. People intend to perform a certain behaviour if their attitude is favourable, if they think other people would approve of it, and if they believe they have the necessary resources and opportunities available [15]. In this sense, knowledge is necessary but might not be sufficient if the attitude is not favourable [16]; the existence of barriers, such as lack of staff, equipment or patient conditions, also needs to be taken into account [17].

According to the framework of Azjen, the attitudes constitute latent and hypothetical dispositions that may interfere with different observable responses that can be collected by direct observation or self-reports [15]. In the area of PI prevention, a recent literature review has described several questionnaires to measure the attitudes [18]. The two validated instruments are Attitudes to pressure ulcer prevention by Moore and Price [19] and Attitude toward Pressure Ulcer Prevention (APuP) by Beeckman et al. [20]. The APuP instrument was chosen because it was developed more recently than others (in 2010) and has been used in a greater number of studies published over the past decade, so this is the instrument that has been better validated [18]. These questionnaires were developed in English, although the APuP has been translated into other languages [21]; however, there is no version available for Spanish-speaking countries.

### 1.2. Aims

The main purpose of our research was to obtain a Spanish version of the APuP and to test its psychometric properties as a first step to measure attitudes to PI prevention in Spanish nurses. A secondary aim was to explore the association of the attitude on PI prevention, as measured by the APuP, with other factors, such as education, knowledge, perceived barriers and nurses’ perceptions. This article reports the results on the validation of the APuP questionnaire, although this is a part of a larger research project aimed at exploring the perception on patient safety, the knowledge on PI prevention, the attitudes to prevention and the perceived barriers by nurses in hospitals in Spain (SECOACBA project) that is currently ongoing in seven hospitals in Spain.

## 2. Materials and Methods

### 2.1. Design

This is an instrument validation study developed in two stages: (1) Translation and cultural adaptation; (2) Psychometric evaluation.

### 2.2. Instrument

The instrument used in this research was the Attitude towards Pressure ulcer Prevention (APuP), developed by Beeckman et al. [20]. The original questionnaire has 13 items grouped into 5 factors, namely: 1-Personal competency to prevent pressure ulcers (3 items), 2-Priority of pressure ulcer prevention (3 items), 3-Impact of pressure ulcers (3 items), 4-Responsibility in pressure ulcer prevention (2 items), 5-Confidence in the effectiveness of prevention (2 items). A 4-point scale is used to answer each item; namely, 1 = strongly disagree, 2 = disagree, 3 = agree, 4 = strongly agree. Some items have to be inversely scored.

The APuP has high content validity (CVI from 0.87 to 1.0), good internal consistency (Cronbach’s alpha = 0.79) and high stability (ICC = 0.89). Higher scores indicate a more positive attitude. Beeckman proposed that a score > 75% of the maximum score can be considered as a satisfactory attitude towards preventing pressure injuries [16].

### 2.3. Procedure

First, permission to translate the questionnaire was obtained from D. Beeckman. Translation and cultural adaptation were carried out according to the usual methodology [22,23]. Briefly, each item was independently translated into Spanish by two bilingual academics with expertise in wound management. Both translations were compared, and minor differences were modified by consensus to reach a single set of items. This version was back-translated into English by a different bilingual expert and then compared, item by item, with the original version; any semantic or conceptual discrepancy was analysed and the Spanish wording revised. The cultural adaption was made over the first Spanish version by two experts in wound care, carefully reading each item to check if they were adequate for the Spanish culture and making some minor changes in the wording, if necessary. After the process, a Spanish version of the APuP questionnaire was obtained and used to determine the psychometric properties (Table 1).

### 2.4. Psychometric Testing: Population

The participants in this study were Registered Nurses (RNs) and Assistant Nurses (ANs) from four hospitals (two acute, one mother and child and one long-term care) from the University Hospital of Jaén (Jaén, Spain). These are public hospitals managed by the Andalusian Health Service.

The inclusion criterion was to have more than 6 months of clinical experience. The sample size was estimated in a minimum of 65 participants (5 people per item of the questionnaire), according to the usual methodological recommendation [24]. Nevertheless, all RNs and ANs working in 29 units of the 4 hospitals were invited to participate and complete the questionnaire, to maximise the sample size of the whole study and reach greater statistical power. Data were collected between March and April 2017.

### 2.5. Data Collection

The instruments used to collect data were as follows:

- The APuP questionnaire, in the new developed Spanish version;
- A brief questionnaire for demographic and educational data;
- A questionnaire with two questions for scoring the perception of patient safety culture and the PI as an adverse effect of hospital stay, both using a scale from 0 to 10 points.

In addition, two additional questionnaires were used in the main study:

- The Pressure Injury Prevention Knowledge questionnaire, in Spanish [13];
- The Pressure Injury Prevention Barriers questionnaire, in Spanish [25].

The set of questionnaires in the paper were provided to participants with an explanatory letter and an envelope for introducing the completed questionnaires. It was stated that the consent to participate was given if a person filled out and delivered it in a sealed envelope.

### 2.6. Ethics

This project was approved by the Committee of Research Ethics of Jaen (number: 2016/12–15). It was also authorised by the direction of the University Hospital, and all nursing unit managers were informed and asked to collaborate. Informed consent was given by participants when completing the questionnaires. No personal data were recorded, and anonymity was guaranteed according to the Spanish Law of Personal Data Protection.

### 2.7. Data Analysis

The data collected were tabulated, coded and cleaned in a spreadsheet prior to analysis. We used the following methods: descriptive statistics (frequencies, percentages, mean and standard deviation), internal consistency by Cronbach’s alpha, confirmatory factor analysis (CFA) and construct validity by the known-groups test with Mann–Whitney test (non-parametric tests were used because the data did not adjust to a normal distribution)**.** The statistical analyses were performed using the software SPSS (IBM Corp, Armonk, NY, USA) and MPlus 7 (Muthén & Muthén, Los Angeles, CA, USA) [26].

#### 2.7.1. Reliability

The internal consistency of the APuP questionnaire was estimated by item-total correlation and Cronbach’s alpha (overall and for each factor).

#### 2.7.2. Validity

##### Confirmatory Factor Analysis

The CFA is a technique to check whether the observed data fit a factor structure previously proposed for the questionnaire. The model used was the 5-factor structure proposed for APuP by Beeckman [20]. The CFA was performed by a structural equation analysis with MPlus. The Weighted Least Squares with Mean and Variance adjustment (WLSMV) method was used to estimate the parameters because the items were ordinal variables, with four options.

Several fit indices were calculated to evaluate the model, according to the methodological recommendations [27,28,29].

- Relative Chi-square (Chi-square divided by the degrees of freedom). Values less than 3 indicate good fit, values between 3 and 5 indicate an acceptable fit and values greater than 5 indicate a poor fit.
- CFI (Comparative Fit Index), has values between 0 and 1. Values greater than >0.95 indicate good fit; if >0.90, they are considered acceptable.
- TLI (Tucker Lewis Index), also called NNFI (Non-Normed Fit Index), with a range between 0 and 1. Values >0.95 indicate a good fit.
- RMSEA (Root Mean Square Error of Approximation), which ranges from 0 to 1. >0.10 justifies the rejection of the model, and values <0.06 indicate a good fit.
- WRMR (Weighted Root Mean Square Residual), calculated based on the difference between the correlation matrices proposed by the model and observed in the data. This weighted indicator is suitable for ordinal variables. Lower values indicate a better fit, and a value of <1.0 is considered a good fit.

##### Test in Known Groups

Two groups were created with expected good/poor attitude towards PI prevention based on the scores on the questions about (1) the perception of patient safety culture and (2) considering PIs as an adverse effect of hospital stay. By means of the known-groups test, it was expected to find that the score in the APuP questionnaire was higher in the group with a good attitude.

The hypotheses formulated were as follows:

- Higher scores on the APuP questionnaire in the group of RNs and ANs who score high on the perception of patient safety culture question compared to those who score low.
- Higher scores on the APuP questionnaire in the group of RNs and ANs who score high on the question about considering PIs as an adverse effect compared to those who score low.

These hypotheses were tested through nonparametric two independent groups mean difference test (Mann–Whitney).

## 3. Results

A total of 438 questionnaires were analysed (response rate = 50.8%).

### 3.1. Sample Characteristics

The characteristics of the sample are displayed in Table 2.

### 3.2. Psychometric Characteristics of The APuP Questionnaire, Spanish Version

#### 3.2.1. Reliability: Internal Consistency

The first analysis of the APuP questionnaire with 13 items (Spanish version) showed that all items except one had good item-total correlation (Table 3). The value of Cronbach’s alpha was 0.65. Item 4 (“Too much attention goes to the prevention of pressure ulcers”) had poor item-total correlation, and its deletion slightly improved the alpha value to 0.70; therefore, this item was deleted and a new analysis was done on the 12-item version. The values of Cronbach’s alpha for the whole questionnaire and each factor are shown in Table 4.

#### 3.2.2. Validity

##### Confirmatory Factor Analysis

Four models of the APuP questionnaire were tested by CFA: (a) the original 5-factor model proposed by Beeckman [20]; (b) the Swedish version with four factors [21]; (c) the 1-factor version, to check if it the instrument fit was better as one-dimensional; (d) the new 12 items with the 5-factor version (Table 5). Model A (5 factors, 13 items) showed fit indices slightly inferior to the good fit values. Again, this analysis showed that item 4 had a low correlation within its factor and low covariance with the remaining items. Model B (4 factors, 13 items) and model C (1 factor, 13 items) showed worse fit indices. Finally, model D (5 factors, 12 items), without item 4, showed the best-fit indices, although the error indices (RMSEA and WRMR) were higher than the values recommended for a good fit.

**Figure 1:**
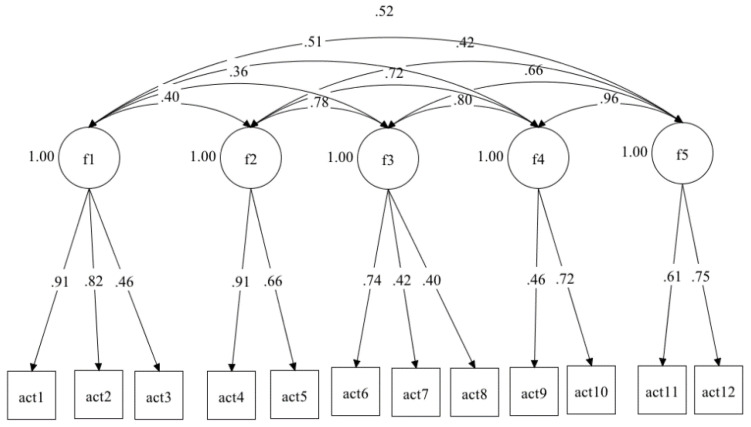
Factor structure of the APuP questionnaire (12-item Spanish version).

The factorial structure of the APuP questionnaire (12-item Spanish version) and the correlations between items and factors are displayed in Figure 1. All factors had high and statistically significant correlations with the overall score in the APuP: factor 1 R = 0.63 (<0.0001); factor 2 R = 0.60 (<0.0001); factor 3 R = 0.68 (<0.0001); factor 4 R = 0.64 (<0.0001) and factor 5 R = 0.57 (<0.0001).

##### Construct Validity

Two hypotheses were tested to check whether the scores obtained with the APuP performed as expected. Table 6 shows the values for the means difference tests; the scores in APuP were higher in the group with high scores both in perception in patient safety culture and in the perception of PI as an adverse effect, but only in the first case, the difference reached statistical significance.

### 3.3. Attitude towards Pressure Ulcer Prevention

The final Spanish version of the APuP questionnaire has 12 items, grouped into 5 factors. Each item can be rated from 1 (Strongly disagree) to 4 (Strongly agree). Items 3, 4, 6, 7, 9 and 12 have to be reserved before scoring. The overall score ranges from 12 to 48 points and is calculated by adding the individual item scores (after reversing the scores of items mentioned before). It is also possible to calculate factors scores: the weighted factor score is the sum of items in the factor divided by the number of items.

In the sample of RNs and ANs from the four hospitals studied, the score with the APuP had a mean of 39.98 (SD 4.18; 95% CI 39.59–40.38). This score is 83.3% of the maximum, which is considered a positive attitude according to the criteria proposed by Beeckman [16]. Table 7 displays the mean scores in each item, sorted from high to low. The four items with the most positive attitude (scores higher than 3.5) refer to the importance and priority of prevention. The two items with a less positive attitude refer to economic aspects.

The factor scores obtained sorted from high to low were (means, DE) F2 Priority 3.59 (0.57), F4 Responsibility 3.38 (0.54), F5 Effectiveness 3.37 (0.53), F3 Impact 3.26 (0.54), F1 Competence 3.21 (0.50).

### 3.4. Associations between APuP Score and Other Factors

An exploratory analysis was made to check if the attitude score with the APuP questionnaire is associated with any professional or educational characteristics (Table 8). Neither the genre nor the work experience are associated with attitude. However, educational factors are associated. The attitude score is slightly higher in RNs than in ANs, and there is a significant difference in the score in people that have received more training on PI prevention versus those with only basic training.

Finally, a correlation analysis between attitude and other variables related to PI prevention was performed. The APuP score has a moderate correlation with the knowledge on prevention (PIPK questionnaire), R = 0.32 (*p* < 0.0001), and a low correlation with perception on patient safety culture, R = 0.15 (*p* = 0.001). No significant correlation was found with PI as adverse effect, R = 0.08 (*p* = 0.08), and with the barriers to PI prevention (BPIP questionnaire), R = −0.08 (*p* = 0.09). However, there was a moderate inverse correlation between the score in the factor Personal competency of the APuP with the score in the barrier questionnaire, R = −0.31 (*p* < 0.0001).

## 4. Discussion

The purpose of this study was to develop a Spanish version of the Attitude toward Pressure ulcer Prevention instrument and to examine its psychometric properties. In addition, it aimed to test the existence of an association between the attitude and other factors, such as the perception of patient safety, knowledge about prevention and perceived barriers to prevention. The APuP questionnaire in the Spanish version with 12 items showed good internal consistency and fit properly in a 5-factor structure.

The development of a new questionnaire requires time and effort, and therefore, it is worthwhile to translate and adapt a questionnaire developed in another language whenever possible. This strategy has the additional advantage that the measurements can be compared. The objective of adapting a questionnaire to another culture is to achieve an instrument equivalent to the original one and with psychometric characteristics (validity and reliability) tested in the place where it is to be used [30].

As the APuP questionnaire was developed, it has been used in many studies in different countries, but not all studies have analysed the psychometric properties of the questionnaire. Reliability is not a fixed feature of a questionnaire or scale, but it depends on the context and population in which it is used. It should therefore be assessed and described in each study [31]. A recent literature review shows a range of internal consistency reliability between 0.63–0.88 for the APuP [18], although some studies do not evaluate their reliability in the population analysed [32,33,34]. For the APuP in the Spanish version, we found a moderate internal consistency (α = 0.70), quite similar to that found in other studies in China (α = 0.69) [35], Turkey (α = 0.66) [36], South Korea (α = 0.72) [37] or Iran (α = 0.74) [38]. Taken together, these results show that the concepts underlying the items of the APuP work well for identifying attitudes towards the prevention, regardless of the linguistic or cultural context.

For the Spanish version of the APuP, one of the items of the original questionnaire had to be removed; it was “Too much attention goes to the prevention of pressure ulcers”. This is one of the reverse-scoring items because agreement implies a poor attitude. In the Spanish translation, probably most of the nurses did not understand well the meaning inside the questionnaire because it was inconsistently scored; therefore, it was decided to remove this item, leaving 12 items in the questionnaire. This is a situation similar to the Korean version with 11 items [37].

Our Spanish version fits well with the 5-factor structure proposed by Beeckman et al. [20]. The CFA showed a reasonably good set of fit and error indices, although not a perfect fit. Therefore, our results agree with Kim and Lee for the Korean version [37], but not with the model with four factors proposed for the Swedish version [21]. Some cultural factors might explain a portion of these differences found in Sweden in the factorial structure of the APuP; however, our study contributes to supporting the original 5-factor model.

The lack of association between the APuP score and the score on PI as an adverse effect (construct validity with known-groups test) has two possible explanations: first, the APuP does not properly measure the latent variable “attitude” (construct validity failure); second, there is no real correlation between these two variables. Because, there are other pieces of evidence of construct validity for the APuP, we think that the second explanation is more probable. However, more research is needed to test whether attitude on PI prevention is associated with other factors.

The attitude towards PI prevention in our population of nurses at the University Hospital of Jaén can be considered as positive because it is higher than the 75% of the maximum score in APuP [16]. These results agree with recent studies in nurses [39,40]; however, other authors described a less positive attitude (74.6%) in operating room nurses [32]. An item of the Impact factor (namely, “A pressure ulcer almost never causes discomfort for a patient”) obtained the highest score (3.69), which is consistent with the findings of other studies [21,32,37,41,42]. We would like to highlight that in our RN and AN population, Priority and Responsibility were the factors of the APuP questionnaire with a higher score, while Personal Competency achieved the lowest score. Other authors have described the factors Impact and Confidence in effectiveness as having the highest and lowest scores, respectively [38,43]. The factor Impact of the APuP reflects both the consequences for patients and institutions; if these injuries are considered to have a low impact, preventive interventions may not be properly implemented [20]. Some studies in European countries, such as Sweden, Finland and the UK, found high scores on the factor Priority for PI prevention, which means that nurses consider it important to apply preventive measures in daily care. However, they scored low on Confidence in the effectiveness of prevention [40,41].

Beeckman defined the factor Responsibility as “the perception of who is responsible for pressure ulcer prevention” [20]. We identified a possible cultural pattern across geographical areas. Studies in the Middle East and Turkey showed that Responsibility scored low [44,45], while in Asian countries, this factor scored highest [32,35,37]. We think that this is an important issue to consider because if nurses and other healthcare providers do not believe they have a personal responsibility in PI prevention, then it is less likely that preventive measures are used.

Our results show an association between the attitude score and some educational variables, such as the professional category. The RNs had slightly higher scores than ANs, meaning a more positive attitude. This agrees with the findings reported by Demarré et al. [46], but not with other authors who failed to find a difference in scores between RNs and ANs [21,47]. In our study, nurses (both RNs and ANs) who had received specific training on PI prevention from multiple sources had a more positive attitude than those with no or only basic training. Similarly, the study conducted by Ünver in Turkey, with nurses from surgical units, found higher scores (*p* = 0.017) in the attitude in nurses trained in PI prevention by attending courses/conferences [44]. There is some evidence about the effect of time elapsed from the training; the effect on the attitude is higher when the courses were attended over the last 6 months compared to over 2 years ago (*p* = 0.001) [45]. In contrast, some authors did not find this effect of the training in PI prevention on nurses’ attitudes [42,43].

Clinical experience working as a nurse seems to not affect the attitude toward PI prevention in our population; the scores in APuP are roughly the same for nurses with less than 10 years of experience and for those with more than 31 years. The same was reported in another study in Turkey [36], but recent research found higher scores on attitude for more experienced nurses (10–14 years) than those with less experience (5–9 years) [38]. Again, this is a point that requires more research to establish whether more experienced nurses really do have a more positive attitude toward prevention.

Is there a significant correlation between knowledge and attitude toward PI prevention? Most of the studies conducted during the last decade in different countries say “Yes”; there is a direct correlation with a low to moderate effect, both using the APuP and the Moore and Price questionnaires (range of values of R from 0.20 to 0.41) [16,38,43,48,49]. Our results are consistent with this range because we also found this correlation (R = 0.32). However, some authors reported discordant results: no correlation [46,50] or an inverse correlation (R: −0.37) in ICU nurses [36]. Therefore, there is sufficient evidence that the nurses who have greater and more updated knowledge about the prevention have a more positive attitude, although it remains unexplained why this correlation does not occur in some groups.

Besides knowledge, the existence of correlations between attitude and other factors has been scarcely analysed. In the present study, we found a direct correlation between attitude and patient safety culture, but not with the perception of PIs as an adverse effect. Nurses more concerned about patient safety scored higher on attitude to PI prevention. A recent study conducted in Spain with health professionals found that most of them (87.7%) considered PIs as a serious adverse event [51]. These relations need to be further explored in different settings and with a larger sample.

Finally, the number of studies about the barriers to PI prevention is increasing [18], but only a few look for associations with other factors. In the present study, we found no association between the attitude and the score on barriers to prevention, but an inverse correlation between the score in the factor Personal competency of the APuP and the score in barriers, meaning that professionals that feel more competent perceive fewer barriers to prevention. Similarly, Coyer et al. found a significant correlation (R = 0.43, *p* = 0.002) between the APuP questionnaire and the item “Overcoming barriers in PI prevention”; a more positive attitude indicates a greater perception of overcoming barriers to PI prevention [52].

Our research has some limitations. For the validation of the Spanish version of the APuP, neither the stability of the questionnaire (test-retest) nor the convergent validity with a gold standard tool were evaluated. There is no instrument to measure Attitude to PI prevention that has consensus to be considered as a gold standard for comparison. The study was conducted in hospitals in only one city in Spain and with a non-random sample, which limits the chances of generalising its results. It is possible that more motivated professionals had participated in the survey, which might lead to an overestimation bias. The response rate under 60% should be also considered as a limitation, although for the main objective of the study the final sample size is estimated as large enough. A larger number of hospitals and other settings, such as primary care centres, should be evaluated with the APuP questionnaire to confirm the findings of this study.

This study has implications for both research and practice. For research, this Spanish version of the APuP provides investigators with a validated tool to measure the attitude towards PI prevention in nurses and other healthcare workers and to search for correlations with other factors or changes following interventions. For practice, this instrument is useful for conducting a rapid survey to assess the attitude towards PI prevention in clinical settings and for evaluating the efficacy of intervention programs, with pre-test–post-test designs. In addition, this tool makes it possible for the identification, at the unit or service level, of those groups of staff with a less positive attitude and to develop tailored interventions for improvement.

The prevention of PI is a complex process involving many factors at different levels: individual (adequate knowledge and positive attitude) and institutional (resources and removal of barriers). Nurses often have difficulties in translating a positive attitude into appropriate preventive strategies due to the existence of barriers [53], and therefore, these complex relationships between knowledge, attitude and barriers are still an issue that needs more research.

## 5. Conclusions

The Spanish version of the APuP questionnaire is a valid, reliable and useful tool to measure the attitude toward PI prevention in Spanish-speaking contexts. This version has 12 items grouped into 5 factors, and its psychometric properties are similar to those of the original instrument.

Nursing staff from public hospitals in a city in southern Spain have a positive attitude toward PI prevention, slightly better in RNs than in ANs. Specific training on PI prevention is associated with better attitude. The amount of knowledge on PI prevention correlates with the score in the APuP instrument. This study highlights the need to take into account not only nurses’ knowledge but also the attitude and perceived barriers when planning the implementation of a program for PI prevention in health care settings.

## Figures and Tables

**Table 1:** The Attitude towards Pressure ulcer Prevention (APuP) questionnaire, English and Spanish versions.

English Version (Beeckman et al.)	Spanish Version
1. I feel confident in my ability to prevent pressure ulcers.	1. Tengo confianza en mi habilidad para prevenir las úlceras por presión.
2. I am well trained to prevent pressure ulcers.	2. Tengo buena formación práctica para prevenir las úlceras por presión.
3. Pressure ulcer prevention is too difficult. Others are better than I am.	3. La prevención de las úlceras por presión es demasiado difícil. Otros lo hacen mejor que yo.
4. Too much attention goes to the prevention of pressure ulcers.	4. Se presta demasiada atención a la prevención de las úlceras por presión.
5. Pressure ulcer prevention is not that important.	5. La prevención de las úlceras por presión no es tan importante.
6. Pressure ulcer prevention should be a priority.	6. La prevención de las úlceras por presión debería ser una prioridad.
7. A pressure ulcer almost never causes discomfort to a patient.	7. Una úlcera por presión casi nunca causa malestar a un paciente.
8. The financial impact of pressure ulcers on a patient should not be exaggerated.	8. El impacto económico de las úlceras por presión en los pacientes no se debería exagerar.
9. The financial impact of pressure ulcers on society is high.	9. El impacto económico de las úlceras por presión en la sociedad es alto.
10. I am not responsible if a pressure ulcer develops in my patients.	10. No soy responsable si se desarrolla una úlcera por presión en mis pacientes.
11. I have an important task in pressure ulcer prevention.	11. Tengo un papel importante en la prevención de las úlceras por presión.
12. Pressure ulcers are preventable in high-risk patients.	12. Las úlceras por presión se pueden prevenir en pacientes con alto riesgo.
13. Pressure ulcers are almost never preventable.	13. Las úlceras por presión casi nunca son prevenibles.

**Table 2:** Characteristics of Registered Nurses and Assistant Nurses.

Variable	Frequency ^1^ (%)
Gender	
Female	354 (80.8)
Male	52 (11.9)
Age (years)	
20–30	7 (1.6)
31–40	57 (13.0)
41–50	162 (37.0)
51–60	192 (43.8)
61–69	16 (3.7)
Professional category	
Registered nurse	266 (60.7)
Assistant nurse	161 (36.8)
Academic degree	
Technical training (2 Years)	150 (34.2)
Nursing diploma (3 Years)	228 (52.1)
Nursing degree (4 Years)	27 (6.2)
Bachelor (4 Years)	7 (1.6)
Postgraduate: Master	15 (3.4)
Doctorate	2 (0.5)
Work experience (years)	
<10	30 (6.8)
11–20	124 (28.3)
21–30	176 (40.2)
>31	104 (23.7)
Specific training in the prevention of PIs	
None	67 (15.3)
Basic ^2^	93 (21.2)
Multiple ^3^	278 (63.5)

^1^ The percentage missing from the variables up to 100% corresponds to the “No” answers. ^2^ Include only basic or undergraduate. ^3^ Multiple training: basic plus conference attendance and/or continuous training.

**Table 3:** Internal consistency reliability of the Attitude towards Pressure Ulcers (Spanish version).

	Version Initial (13 Items)		Version Modified (12 Items)	
Item	Item-Total Correlation	Cronbach’s Alpha (If Deleted)	Item-Total Correlation	Cronbach’s Alpha (If Deleted)
1	0.34	0.63	0.38	0.66
2	0.27	0.64	0.31	0.67
3	0.34	0.63	0.32	0.67
4	0.05	0.70	Deleted	Deleted
5	0.41	0.62	0.40	0.66
6	0.34	0.63	0.34	0.67
7	0.29	0.63	0.30	0.67
8	0.21	0.65	0.24	0.69
9	0.24	0.64	0.25	0.68
10	0.30	0.63	0.29	0.67
11	0.39	0.62	0.42	0.66
12	0.28	0.64	0.31	0.67
13	0.44	0.61	0.44	0.66

**Table 4:** Internal consistency reliability of the Attitude towards Pressure Ulcers (Spanish version).

	Alpha Cronbach
Global (12 items)	0.70
Factor 1 (3 items)	0.58
Factor 2 (2 items)	0.58
Factor 3 (3 items)	0.32
Factor 4 (2 items)	0.33
Factor 5 (2 items)	0.51

**Table 5:** Confirmatory factor analysis of several models of the APuP questionnaire (Spanish version).

	Relative Chi-Square	CFI	TLI	RMSEA (IC95%)	WRMR
Good fit values	<3	>0.95	>0.95	<0.06	<1.0
Model A (5 factors-13 items)	3.63	0.93	0.90	0.08 (0.07–0.09)	1.20
Model B (4 factors-3 items)	4.53	0.89	0.86	0.09 (0.08–0.10)	1.44
Model C (1 factor-13 items)	7.50	0.79	0.74	0.12 (0.11–0.13)	2.01
Model D (5 factors-12 items)	3.33	0.94	0.92	0.07 (0.06–0.08)	1.13

**Table 6:** Known-group test of the APuP questionnaire (Spanish version).

Variable	Mean (SD)	*p*-Value
Patient safety culture ^1^		
Low	39.3 (3.9)	0.016
High	41.4 (4.2)	
PI as adverse effect ^2^		
Low	40.5 (4.2)	0.601
High	41.0 (4.7)	

SD: Standard deviation. *p* value for groups difference with the Mann–Whitney test. ^1^ Patient safety culture: Low ≤ 6 (percentile 10); High = 10 (percentile 90). ^2^ PI as adverse effect: Low ≤ 3 (percentile 10); High = 10 (percentile 90).

**Table 7:** Descriptive values of the items of the questionnaire of attitudes towards the prevention of LPP (Spanish version).

Item	Mean	Standard Deviation
6. A pressure ulcer almost never causes discomfort for a patient.	3.69	0.70
4. Pressure ulcer prevention is not that important.	3.67	0.68
10. I have an important task in pressure ulcer prevention.	3.58	0.65
5. Pressure ulcer prevention should be a priority.	3.51	0.68
12. Pressure ulcers are almost never preventable.	3.49	0.63
1. I feel confident in my ability to prevent pressure ulcers.	3.35	0.57
11. Pressure ulcers are preventable in high-risk patients.	3.26	0.70
9. I am not responsible if a pressure ulcer develops in my patients.	3.20	0.75
2. I am well trained to prevent pressure ulcers.	3.18	0.63
3. Pressure ulcer prevention is too difficult. Others are better than I am.	3.11	0.78
8. The financial impact of pressure ulcers on society is high.	3.08	0.82
7. The financial impact of pressure ulcers on a patient should not be exaggerated.	3.02	0.96

**Table 8:** Attitude score with APuP questionnaire in different subgroups.

Variable	APuP Score Mean (SD)	*p*-Value ^1^
**Gender**		
Male	39.65 (4.27)	0.375
Female	40.20 (4.04)	
Professional category		
Registered nurse	40.55 (3.98)	0.001
Assistant nurse	39.08 (4.45)	
Work Experience (years)		
<10	40.70 (3.42)	0.484
11–20	39.60 (4.44)	
21–30	40.25 (4.18)	
>31	39.79 (4.11)	
Specific training in prevention of PIs		
None	38.95 (3.53)	*p* < 0.0001
Basic	38.63 (4.88)	
Multiple ^2^	40.68 (3.92)	

^1^ Mann-Whitney or Kruskal–Wallis test. ^2^ Multiple training: basic plus conference attendance and/or continuous training. Basic/None: No difference; Multiple/None: *p* = 0.001; Multiple/Basic: *p* < 0.0001.
